# Supplementary material for: Resolving the full spectrum of human genome variation using Linked-Reads
Source: Genome Res. 2019 Apr;29(4):635–45. doi: 10.1101/gr.234443.118 (PMC6442396; doi:10.1101/gr.234443.118)
Supplement: Supplemental Material [file supp_gr.234443.118_Supplemental_longranger-2.2.zip › longranger-2.2.tar.bz2!/lr2.2-public/lib/stan/cmdstan/stan/doc/api/html/meta_2traits_8hpp_source.html]

Stan: src/stan/meta/traits.hpp Source File


|  |  |
| --- | --- |
|  | Stan  2.5.0  probability, sampling & optimization |


- Main Page
- Related Pages
- Namespaces
- Classes
- Files

- File List
- File Members

All Classes Namespaces Files Functions Variables Typedefs Enumerations Enumerator Friends Macros Pages

- src
- stan
- meta

traits.hpp

Go to the documentation of this file.

1 #ifndef STAN\_\_META\_\_TRAITS\_HPP

2 #define STAN\_\_META\_\_TRAITS\_HPP

3

4

5 #include <vector>

6 #include <boost/type\_traits.hpp>

7 #include <boost/type\_traits/is\_arithmetic.hpp>

8 #include <boost/math/tools/promotion.hpp>

9

10 #include <stan/agrad/fwd/fvar.hpp>

11 #include <stan/agrad/rev/var.hpp>

12

13 #include <stan/math/matrix/Eigen.hpp>

14 #include <stan/math/matrix/meta/value\_type.hpp>

15 #include <stan/math/meta/value\_type.hpp>

16

17 namespace stan {

18

19  struct error\_index {

20  enum { value =

21 #ifdef ERROR\_INDEX

22 ERROR\_INDEX

23 #else

24 1

25 #endif

26  };

27  };

28

42  template <typename T>

43  struct is\_constant {

48  enum { value = boost::is\_convertible<T,double>::value };

49  };

50

51

56  template <typename T>

57  struct is\_constant\_struct {

58  enum { value = is\_constant<T>::value };

59  };

60

61

62  template <typename T>

63  struct is\_constant\_struct<std::vector<T> > {

64  enum { value = is\_constant\_struct<T>::value };

65  };

66

67  template <typename T, int R, int C>

68  struct is\_constant\_struct<Eigen::Matrix<T,R,C> > {

69  enum { value = is\_constant\_struct<T>::value };

70  };

71

72  template <typename T>

73  struct is\_constant\_struct<Eigen::Block<T> > {

74  enum { value = is\_constant\_struct<T>::value };

75  };

76

77

78

79

80  // FIXME: use boost::type\_traits::remove\_all\_extents to extend to array/ptr types

81

82  template <typename T>

83  struct is\_vector {

84  enum { value = 0 };

85  typedef T type;

86  };

87  template <typename T>

88  struct is\_vector<const T> {

89  enum { value = is\_vector<T>::value };

90  typedef T type;

91  };

92  template <typename T>

93  struct is\_vector<std::vector<T> > {

94  enum { value = 1 };

95  typedef T type;

96  };

97

98  template <typename T>

99  struct is\_vector<Eigen::Matrix<T,Eigen::Dynamic,1> > {

100  enum { value = 1 };

101  typedef T type;

102  };

103  template <typename T>

104  struct is\_vector<Eigen::Matrix<T,1,Eigen::Dynamic> > {

105  enum { value = 1 };

106  typedef T type;

107  };

108  template <typename T>

109  struct is\_vector<Eigen::Block<T> > {

110  enum { value = 1 };

111  typedef T type;

112  };

113

114

115  namespace {

116  template <bool is\_vec, typename T>

117  struct scalar\_type\_helper {

118  typedef T type;

119  };

120

121  template <typename T>

122  struct scalar\_type\_helper<true, T> {

123  typedef typename

124  scalar\_type\_helper<is\_vector<typename stan::math::value\_type<T>::type>::value,

125  typename stan::math::value\_type<T>::type>::type

126  type;

127  };

128  }

137  template <typename T>

138  struct scalar\_type {

139  typedef typename scalar\_type\_helper<is\_vector<T>::value, T>::type type;

140  };

141

142  template <typename T>

143  inline T get(const T& x, size\_t n) {

144  return x;

145  }

146  template <typename T>

147  inline T get(const std::vector<T>& x, size\_t n) {

148  return x[n];

149  }

150  template <typename T, int R, int C>

151  inline T get(const Eigen::Matrix<T,R,C>& m, size\_t n) {

152  return m(static\_cast<int>(n));

153  }

154

155

156

157  // length() should only be applied to primitive or std vector or Eigen vector

158  template <typename T>

159  size\_t length(const T& /\*x\*/) {

160  return 1U;

161  }

162  template <typename T>

163  size\_t length(const std::vector<T>& x) {

164  return x.size();

165  }

166  template <typename T, int R, int C>

167  size\_t length(const Eigen::Matrix<T,R,C>& m) {

168  return m.size();

169  }

170

171  template<typename T, bool is\_vec>

172  struct size\_of\_helper {

173  static size\_t size\_of(const T& /\*x\*/) {

174  return 1U;

175  }

176  };

177

178  template<typename T>

179  struct size\_of\_helper<T, true> {

180  static size\_t size\_of(const T& x) {

181  return x.size();

182  }

183  };

184

185  template <typename T>

186  size\_t size\_of(const T& x) {

187  return size\_of\_helper<T, is\_vector<T>::value>::size\_of(x);

188  }

189

190  template <typename T1, typename T2>

191  size\_t max\_size(const T1& x1, const T2& x2) {

192  size\_t result = length(x1);

193  result = result > length(x2) ? result : length(x2);

194  return result;

195  }

196

197  template <typename T1, typename T2, typename T3>

198  size\_t max\_size(const T1& x1, const T2& x2, const T3& x3) {

199  size\_t result = length(x1);

200  result = result > length(x2) ? result : length(x2);

201  result = result > length(x3) ? result : length(x3);

202  return result;

203  }

204

205  template <typename T1, typename T2, typename T3, typename T4>

206  size\_t max\_size(const T1& x1, const T2& x2, const T3& x3, const T4& x4) {

207  size\_t result = length(x1);

208  result = result > length(x2) ? result : length(x2);

209  result = result > length(x3) ? result : length(x3);

210  result = result > length(x4) ? result : length(x4);

211  return result;

212  }

213

214  // \*\*\*\*\*\*\*\*\*\*\*\*\*\*\*\*\*\* additions for new VV \*\*\*\*\*\*\*\*\*\*\*\*\*\*\*\*\*\*\*\*\*\*\*\*\*

215  template <typename T>

216  struct scalar\_type<Eigen::Matrix<T,Eigen::Dynamic,Eigen::Dynamic> > {

217  typedef typename scalar\_type<T>::type type;

218  };

219

220  template <typename T>

221  struct scalar\_type<T\*> {

222  typedef typename scalar\_type<T>::type type;

223  };

224

225

226  // handles scalar, eigen vec, eigen row vec, std vec

227  template <typename T>

228  struct is\_vector\_like {

229  enum { value = stan::is\_vector<T>::value };

230  };

231  template <typename T>

232  struct is\_vector\_like<T\*> {

233  enum { value = true };

234  };

235  // handles const

236  template <typename T>

237  struct is\_vector\_like<const T> {

238  enum { value = stan::is\_vector\_like<T>::value };

239  };

240  // handles eigen matrix

241  template <typename T>

242  struct is\_vector\_like<Eigen::Matrix<T,Eigen::Dynamic,Eigen::Dynamic> > {

243  enum { value = true };

244  };

245

246

272  template <typename T,

273  bool is\_array = stan::is\_vector\_like<T>::value,

274  bool throw\_if\_accessed = false>

275  class VectorView {

276  public:

277  typedef typename scalar\_type<T>::type scalar\_t;

278

279  VectorView(scalar\_t& c) : x\_(&c) { }

280

281  VectorView(std::vector<scalar\_t>& v) : x\_(&v[0]) { }

282

283  template <int R, int C>

284  VectorView(Eigen::Matrix<scalar\_t,R,C>& m) : x\_(&m(0)) { }

285

286  VectorView(scalar\_t\* x) : x\_(x) { }

287

288  scalar\_t& operator[](int i) {

289  if (throw\_if\_accessed)

290  throw std::out\_of\_range("VectorView: this cannot be accessed");

291  if (is\_array)

292  return x\_[i];

293  else

294  return x\_[0];

295  }

296  private:

297  scalar\_t\* x\_;

298  };

299

304  template <typename T, bool is\_array, bool throw\_if\_accessed>

305  class VectorView<const T, is\_array, throw\_if\_accessed> {

306  public:

307  typedef typename scalar\_type<T>::type scalar\_t;

308

309  VectorView(const scalar\_t& c) : x\_(&c) { }

310

311  VectorView(const scalar\_t\* x) : x\_(x) { }

312

313  VectorView(const std::vector<scalar\_t>& v) : x\_(&v[0]) { }

314

315  template <int R, int C>

316  VectorView(const Eigen::Matrix<scalar\_t,R,C>& m) : x\_(&m(0)) { }

317

318  const scalar\_t& operator[](int i) const {

319  if (throw\_if\_accessed)

320  throw std::out\_of\_range("VectorView: this cannot be accessed");

321  if (is\_array)

322  return x\_[i];

323  else

324  return x\_[0];

325  }

326  private:

327  const scalar\_t\* x\_;

328  };

329

330  // simplify to hold value in common case where it's more efficient

331  template <>

332  class VectorView<const double, false, false> {

333  public:

334  VectorView(double x) : x\_(x) { }

335  double operator[](int /\* i \*/) const {

336  return x\_;

337  }

338  private:

339  const double x\_;

340  };

341

342

357  template<bool used, bool is\_vec>

358  class DoubleVectorView {

359  public:

360  DoubleVectorView(size\_t /\* n \*/) { }

361  double& operator[](size\_t /\* i \*/) {

362  throw std::runtime\_error("used is false. this should never be called");

363  }

364  };

365

366  template<>

367  class DoubleVectorView<true, false> {

368  private:

369  double x\_;

370  public:

371  DoubleVectorView(size\_t /\* n \*/) : x\_(0.0) { }

372  double& operator[](size\_t /\* i \*/) {

373  return x\_;

374  }

375  };

376

377  template<>

378  class DoubleVectorView<true, true> {

379  private:

380  std::vector<double> x\_;

381  public:

382  DoubleVectorView(size\_t n) : x\_(n) { }

383  double& operator[](size\_t i) {

384  return x\_[i];

385  }

386  };

387

392  template <typename T1,

393  typename T2 = double,

394  typename T3 = double,

395  typename T4 = double,

396  typename T5 = double,

397  typename T6 = double>

398  struct return\_type {

399  typedef typename

400  boost::math::tools::promote\_args<typename scalar\_type<T1>::type,

401  typename scalar\_type<T2>::type,

402  typename scalar\_type<T3>::type,

403  typename scalar\_type<T4>::type,

404  typename scalar\_type<T5>::type,

405  typename scalar\_type<T6>::type>::type

406  type;

407  };

408

409

410  template <typename T>

411  struct is\_fvar {

412  enum { value = false };

413  };

414  template <typename T>

415  struct is\_fvar<stan::agrad::fvar<T> > {

416  enum { value = true };

417  };

418

419

420  template <typename T>

421  struct is\_var {

422  enum { value = false };

423  };

424  template <>

425  struct is\_var<stan::agrad::var> {

426  enum { value = true };

427  };

428

429

430

431  // FIXME: pull out scalar types

432

437  template <typename T1,

438  typename T2 = double,

439  typename T3 = double,

440  typename T4 = double,

441  typename T5 = double,

442  typename T6 = double>

443  struct contains\_fvar {

444  enum {

445  value = is\_fvar<typename scalar\_type<T1>::type>::value

446  || is\_fvar<typename scalar\_type<T2>::type>::value

447  || is\_fvar<typename scalar\_type<T3>::type>::value

448  || is\_fvar<typename scalar\_type<T4>::type>::value

449  || is\_fvar<typename scalar\_type<T5>::type>::value

450  || is\_fvar<typename scalar\_type<T6>::type>::value

451  };

452  };

453

454

455  template <typename T1,

456  typename T2 = double,

457  typename T3 = double,

458  typename T4 = double,

459  typename T5 = double,

460  typename T6 = double>

461  struct is\_var\_or\_arithmetic {

462  enum {

463  value

464  = (is\_var<typename scalar\_type<T1>::type>::value

465  || boost::is\_arithmetic<typename scalar\_type<T1>::type>::value)

466  && (is\_var<typename scalar\_type<T2>::type>::value

467  || boost::is\_arithmetic<typename scalar\_type<T2>::type>::value)

468  && (is\_var<typename scalar\_type<T3>::type>::value

469  || boost::is\_arithmetic<typename scalar\_type<T3>::type>::value)

470  && (is\_var<typename scalar\_type<T4>::type>::value

471  || boost::is\_arithmetic<typename scalar\_type<T4>::type>::value)

472  && (is\_var<typename scalar\_type<T5>::type>::value

473  || boost::is\_arithmetic<typename scalar\_type<T5>::type>::value)

474  && (is\_var<typename scalar\_type<T6>::type>::value

475  || boost::is\_arithmetic<typename scalar\_type<T6>::type>::value)

476  };

477  };

478

479  namespace {

480  template <bool is\_vec, typename T, typename T\_container>

481  struct scalar\_type\_helper\_pre {

482  typedef T\_container type;

483  };

484

485  template <typename T, typename T\_container>

486  struct scalar\_type\_helper\_pre<true, T, T\_container> {

487  typedef typename

488  scalar\_type\_helper\_pre<is\_vector<typename stan::math::value\_type<T>::type>::value,

489  typename stan::math::value\_type<T>::type,

490  typename stan::math::value\_type<T\_container>::type>::type

491  type;

492  };

493  }

494

501  template <typename T>

502  struct scalar\_type\_pre {

503  typedef typename

504  scalar\_type\_helper\_pre<is\_vector<typename stan::math::value\_type<T>::type>::value,

505  typename stan::math::value\_type<T>::type, T>::type

506  type;

507  };

508

509

510  template <typename T,

511  bool is\_array

512  = stan::is\_vector\_like<typename stan::math::value\_type<T>::type>::value,

513  bool throw\_if\_accessed = false>

514  class VectorViewMvt {

515  public:

516  typedef typename scalar\_type\_pre<T>::type matrix\_t;

517

518  VectorViewMvt(matrix\_t& m) : x\_(&m) { }

519

520  VectorViewMvt(std::vector<matrix\_t>& vm) : x\_(&vm[0]) { }

521

522  matrix\_t& operator[](int i) {

523  if (throw\_if\_accessed)

524  throw std::out\_of\_range("VectorViewMvt: this cannot be accessed");

525  if (is\_array)

526  return x\_[i];

527  else

528  return x\_[0];

529  }

530  private:

531  matrix\_t\* x\_;

532  };

533

538  template <typename T, bool is\_array, bool throw\_if\_accessed>

539  class VectorViewMvt<const T, is\_array, throw\_if\_accessed> {

540  public:

541  typedef typename scalar\_type\_pre<T>::type matrix\_t;

542

543  VectorViewMvt(const matrix\_t& m) : x\_(&m) { }

544

545  VectorViewMvt(const std::vector<matrix\_t>& vm) : x\_(&vm[0]) { }

546

547  const matrix\_t& operator[](int i) const {

548  if (throw\_if\_accessed)

549  throw std::out\_of\_range("VectorViewMvt: this cannot be accessed");

550  if (is\_array)

551  return x\_[i];

552  else

553  return x\_[0];

554  }

555  private:

556  const matrix\_t\* x\_;

557  };

558

559  // length\_mvt() should only be applied to std vector or Eigen matrix

560  template <typename T>

561  size\_t length\_mvt(const T& ) {

562  throw std::out\_of\_range("length\_mvt passed to an unrecognized type.");

563  return 1U;

564  }

565  template <typename T, int R, int C>

566  size\_t length\_mvt(const Eigen::Matrix<T,R,C>& ) {

567  return 1U;

568  }

569  template <typename T, int R, int C>

570  size\_t length\_mvt(const std::vector<Eigen::Matrix<T,R,C> >& x) {

571  return x.size();

572  }

573

574  template <typename T1, typename T2>

575  size\_t max\_size\_mvt(const T1& x1, const T2& x2) {

576  size\_t result = length\_mvt(x1);

577  result = result > length\_mvt(x2) ? result : length\_mvt(x2);

578  return result;

579  }

580

581  template <typename T1, typename T2, typename T3>

582  size\_t max\_size\_mvt(const T1& x1, const T2& x2, const T3& x3) {

583  size\_t result = length\_mvt(x1);

584  result = result > length\_mvt(x2) ? result : length\_mvt(x2);

585  result = result > length\_mvt(x3) ? result : length\_mvt(x3);

586  return result;

587  }

588

589  template <typename T1, typename T2, typename T3, typename T4>

590  size\_t max\_size\_mvt(const T1& x1, const T2& x2, const T3& x3, const T4& x4) {

591  size\_t result = length\_mvt(x1);

592  result = result > length\_mvt(x2) ? result : length\_mvt(x2);

593  result = result > length\_mvt(x3) ? result : length\_mvt(x3);

594  result = result > length\_mvt(x4) ? result : length\_mvt(x4);

595  return result;

596  }

597

598 }

599 #endif

600

stan::is\_vector< Eigen::Block< T > >::type

T type

**Definition:** traits.hpp:111

stan::scalar\_type< T \* >::type

scalar\_type< T >::type type

**Definition:** traits.hpp:222

stan::is\_constant

Metaprogramming struct to detect whether a given type is constant in the mathematical sense (not the ...

**Definition:** traits.hpp:43

stan::DoubleVectorView< true, true >::operator[]

double & operator[](size\_t i)

**Definition:** traits.hpp:383

stan::scalar\_type

Metaprogram structure to determine the base scalar type of a template argument.

**Definition:** traits.hpp:138

stan::VectorView::operator[]

scalar\_t & operator[](int i)

**Definition:** traits.hpp:288

stan::VectorView::VectorView

VectorView(scalar\_t &c)

**Definition:** traits.hpp:279

stan::max\_size\_mvt

size\_t max\_size\_mvt(const T1 &x1, const T2 &x2)

**Definition:** traits.hpp:575

stan::VectorView::scalar\_t

scalar\_type< T >::type scalar\_t

**Definition:** traits.hpp:277

stan::is\_constant::value

**Definition:** traits.hpp:48

stan::DoubleVectorView::operator[]

double & operator[](size\_t)

**Definition:** traits.hpp:361

stan::size\_of\_helper::size\_of

static size\_t size\_of(const T &)

**Definition:** traits.hpp:173

stan::scalar\_type\_pre::type

scalar\_type\_helper\_pre< is\_vector< typename stan::math::value\_type< T >::type >::value, typename stan::math::value\_type< T >::type, T >::type type

**Definition:** traits.hpp:506

stan::is\_vector\_like

**Definition:** traits.hpp:228

stan::is\_vector::type

T type

**Definition:** traits.hpp:85

stan::size\_of\_helper< T, true >::size\_of

static size\_t size\_of(const T &x)

**Definition:** traits.hpp:180

stan::is\_vector::value

**Definition:** traits.hpp:84

stan::DoubleVectorView< true, true >::DoubleVectorView

DoubleVectorView(size\_t n)

**Definition:** traits.hpp:382

stan::is\_var

**Definition:** traits.hpp:421

stan::DoubleVectorView< true, false >::operator[]

double & operator[](size\_t)

**Definition:** traits.hpp:372

stan::is\_vector< std::vector< T > >::type

T type

**Definition:** traits.hpp:95

stan::is\_vector\_like::value

**Definition:** traits.hpp:229

stan::VectorViewMvt::VectorViewMvt

VectorViewMvt(std::vector< matrix\_t > &vm)

**Definition:** traits.hpp:520

stan::agrad::fvar

**Definition:** fvar.hpp:13

stan::is\_var\_or\_arithmetic::value

**Definition:** traits.hpp:463

stan::length

size\_t length(const T &)

**Definition:** traits.hpp:159

stan::return\_type

Metaprogram to calculate the base scalar return type resulting from promoting all the scalar types of...

**Definition:** traits.hpp:398

stan::DoubleVectorView

DoubleVectorView allocates double values to be used as intermediate values.

**Definition:** traits.hpp:358

stan::is\_constant\_struct::value

**Definition:** traits.hpp:58

stan::scalar\_type::type

scalar\_type\_helper< is\_vector< T >::value, T >::type type

**Definition:** traits.hpp:139

stan::VectorViewMvt< const T, is\_array, throw\_if\_accessed >::VectorViewMvt

VectorViewMvt(const std::vector< matrix\_t > &vm)

**Definition:** traits.hpp:545

stan::is\_vector

**Definition:** traits.hpp:83

stan::return\_type::type

boost::math::tools::promote\_args< typename scalar\_type< T1 >::type, typename scalar\_type< T2 >::type, typename scalar\_type< T3 >::type, typename scalar\_type< T4 >::type, typename scalar\_type< T5 >::type, typename scalar\_type< T6 >::type >::type type

**Definition:** traits.hpp:406

stan::VectorViewMvt::operator[]

matrix\_t & operator[](int i)

**Definition:** traits.hpp:522

stan::VectorView::VectorView

VectorView(std::vector< scalar\_t > &v)

**Definition:** traits.hpp:281

stan::is\_constant\_struct

Metaprogram to determine if a type has a base scalar type that can be assigned to type double...

**Definition:** traits.hpp:57

stan::error\_index::value

**Definition:** traits.hpp:20

stan::VectorView< const T, is\_array, throw\_if\_accessed >::VectorView

VectorView(const scalar\_t \*x)

**Definition:** traits.hpp:311

stan::VectorView< const T, is\_array, throw\_if\_accessed >::scalar\_t

scalar\_type< T >::type scalar\_t

**Definition:** traits.hpp:307

stan::is\_fvar

**Definition:** traits.hpp:411

stan::size\_of\_helper

**Definition:** traits.hpp:172

stan::VectorView< const double, false, false >::VectorView

VectorView(double x)

**Definition:** traits.hpp:334

fvar.hpp

value\_type.hpp

stan::size\_of

size\_t size\_of(const T &x)

**Definition:** traits.hpp:186

stan::VectorView< const T, is\_array, throw\_if\_accessed >::VectorView

VectorView(const std::vector< scalar\_t > &v)

**Definition:** traits.hpp:313

value\_type.hpp

stan::length\_mvt

size\_t length\_mvt(const T &)

**Definition:** traits.hpp:561

var.hpp

stan::scalar\_type< Eigen::Matrix< T, Eigen::Dynamic, Eigen::Dynamic > >::type

scalar\_type< T >::type type

**Definition:** traits.hpp:217

stan::max\_size

size\_t max\_size(const T1 &x1, const T2 &x2)

**Definition:** traits.hpp:191

stan::is\_fvar::value

**Definition:** traits.hpp:412

stan::VectorView::VectorView

VectorView(Eigen::Matrix< scalar\_t, R, C > &m)

**Definition:** traits.hpp:284

Eigen.hpp

stan::is\_vector< Eigen::Matrix< T, Eigen::Dynamic, 1 > >::type

T type

**Definition:** traits.hpp:101

stan::VectorView< const T, is\_array, throw\_if\_accessed >::operator[]

const scalar\_t & operator[](int i) const

**Definition:** traits.hpp:318

stan::VectorView< const double, false, false >::operator[]

double operator[](int) const

**Definition:** traits.hpp:335

stan::agrad::var

Independent (input) and dependent (output) variables for gradients.

**Definition:** var.hpp:27

stan::VectorViewMvt< const T, is\_array, throw\_if\_accessed >::operator[]

const matrix\_t & operator[](int i) const

**Definition:** traits.hpp:547

stan::VectorViewMvt::VectorViewMvt

VectorViewMvt(matrix\_t &m)

**Definition:** traits.hpp:518

stan::is\_vector< const T >::type

T type

**Definition:** traits.hpp:90

stan::DoubleVectorView::DoubleVectorView

DoubleVectorView(size\_t)

**Definition:** traits.hpp:360

stan::is\_var::value

**Definition:** traits.hpp:422

stan::VectorViewMvt

**Definition:** traits.hpp:514

stan::is\_var\_or\_arithmetic

**Definition:** traits.hpp:461

stan::VectorView< const T, is\_array, throw\_if\_accessed >::VectorView

VectorView(const scalar\_t &c)

**Definition:** traits.hpp:309

stan::VectorViewMvt::matrix\_t

scalar\_type\_pre< T >::type matrix\_t

**Definition:** traits.hpp:516

stan::VectorViewMvt< const T, is\_array, throw\_if\_accessed >::VectorViewMvt

VectorViewMvt(const matrix\_t &m)

**Definition:** traits.hpp:543

stan::scalar\_type\_pre

Metaprogram structure to determine the type of first container of the base scalar type of a template ...

**Definition:** traits.hpp:502

stan::contains\_fvar

Metaprogram to calculate the base scalar return type resulting from promoting all the scalar types of...

**Definition:** traits.hpp:443

stan::is\_vector< Eigen::Matrix< T, 1, Eigen::Dynamic > >::type

T type

**Definition:** traits.hpp:106

stan::error\_index

**Definition:** traits.hpp:19

stan::VectorView::VectorView

VectorView(scalar\_t \*x)

**Definition:** traits.hpp:286

stan::VectorView

VectorView is a template metaprogram that takes its argument and allows it to be used like a vector...

**Definition:** traits.hpp:275

stan::VectorView< const T, is\_array, throw\_if\_accessed >::VectorView

VectorView(const Eigen::Matrix< scalar\_t, R, C > &m)

**Definition:** traits.hpp:316

stan::DoubleVectorView< true, false >::DoubleVectorView

DoubleVectorView(size\_t)

**Definition:** traits.hpp:371

stan::VectorViewMvt< const T, is\_array, throw\_if\_accessed >::matrix\_t

scalar\_type\_pre< T >::type matrix\_t

**Definition:** traits.hpp:541

stan::math::value\_type

Primary template class for metaprogram to compute the type of values stored in a container.

**Definition:** value\_type.hpp:21

stan::contains\_fvar::value

**Definition:** traits.hpp:445


---

[ Stan Home Page ]

*© 2011–2014,
Stan Development Team.*
